# Assessment of sleep problems in patients with Kawasaki disease: a survey-based study

**DOI:** 10.1186/s12887-025-05418-w

**Published:** 2025-02-04

**Authors:** Samar Tharwat, Mohammed Kamal Nassar

**Affiliations:** 1 Rheumatology & Immunology Unit, Department of Internal Medicine, Faculty of Medicine, Mansoura University, Mansoura, Egypt; 2 Department of Internal Medicine, Faculty of Medicine, Horus University, New Damietta, Egypt; 3 Mansoura Nephrology and Dialysis Unit, Department of Internal Medicine, Faculty of Medicine, Mansoura University, Mansoura, Egypt; 4 Mansoura University Hospital, El Gomhouria St, Mansoura, Dakahlia Governorate 35511 Egypt

**Keywords:** Children’s sleep habits questionnaire, Sleep, Kawasaki disease, Survey

## Abstract

**Introduction:**

Kawasaki disease (KD) is a rare systemic inflammatory disease that primarily affects children under the age of five. It is now recognized as the most prevalent cause of acquired heart diseases in children in developed countries.

**Objectives:**

The aim of this study was to evaluate sleep disturbances in patients with KD and identify their prevalence and associations.

**Methods:**

This cross-sectional analytic survey-based study was carried out on 262 participants (130 KD patients and 132 age- and sex-matched healthy controls). Participants were invited via social media groups. Sociodemographic data, clinical characteristics and therapeutic data of KD patients were collected. To identify the presence of sleep disturbances, all participants completed Children’s Sleep Habits Questionnaire (CSHQ).

**Results:**

The median age for KD patients was 6 years, and 51.5% of them were female, the median age at disease onset was 2.5 years, and the median disease duration was 3 years. The sleep scores of patients with KD were significantly higher than those of the control group (55.72 ± 11.97 vs. 49.45 ± 8.54, *p* < 0.001). The total duration of sleep did not exhibit any statistically significant difference between patients with KD and healthy controls (*p* = 0.399). KD patients exhibited significantly elevated scores in sleep onset delay, sleep duration, night wakings, parasomnias, and sleep-disordered breathing (*p* < 0.001). Additionally, they showed marginally higher scores in daytime sleepiness (*p* = 0.059). Younger age of KD patients was associated with higher rates of bedtime resistance (*p* < 0.001) and sleep anxiety (*p* = 0.005). Younger age at KD onset was associated significantly with higher rates of bedtime resistance (*p* = 0.009), sleep anxiety (*p* = 0.038), night wakings (*p* = 0.017), and worse sleep quality (*p* = 0.033). KD Patients who exhibited lethargy, and received corticosteroid medication had significantly higher sleep scores than those who did not.

**Conclusion:**

Patients with KD experience higher sleep disturbance than their healthy counterparts. Young age, early disease onset, lethargy and corticosteroid administration are linked to poor sleep quality.

## Introduction

Kawasaki disease (KD) is a rare inflammatory condition that primarily affects children under 5 years old [1]. It is pathologically characterized by systemic vasculitis, which primarily affects medium-sized arteries [2]. KD is a worldwide illness that is characterized by varying incidence rates that are predominantly influenced by the racial composition of the populations of different countries. Japan has the highest incidence of KD, which has consistently increased at an annual rate of 308 per 100,000 children under the age of five in 2014 [3]. The male-to-female ratio of KD patients is approximately 1.5 to 1 in almost all countries [3]. It is the most prevalent type of childhood primary vasculitis worldwide. Failure to promptly diagnose and treat the condition leads to the development of coronary artery aneurysms in around 25% of all affected individuals [2]. Lymphadenopathy, fever, and mucocutaneous changes are the common symptoms that accompany this disease. However, KD is an exceptional disease in which incomplete variants of the disease are prevalent and unusual manifestations are frequently observed [4]. Currently, high-dose intravenous immune globulin (IVIG) and aspirin (ASA) are the cornerstones of treatment [5].

Sleep is crucial for maintaining good health and well-being, particularly during the early stages of life. Lack of sleep has been linked to negative effects on brain development, cognitive abilities, emotional control, depression, and obesity [6]. Additionally, unresolved sleep difficulties that persist for months can be stressful for families and impede daily activities [7]. Healthy sleep depends critically on enough duration, good quality, regularity, and absence of sleep disturbances [8]. Sleep disturbances are significantly more common among medically ill children and adolescents compared to the general population. The existence of comorbidity might have a negative impact on the medical outcomes and health related quality of life of these patients [9]. Patients with KD reported experiencing a variety of sleep-related symptoms, such as sweating, sleeplessness, stuffy noses, or awakening with energy that would not allow sleep [10].

One of the most critical steps toward the development of strategies and interventions to reduce sleep disturbance and the unfavorable health effects that it causes is the characterization of sleep patterns and the identification of factors that affect sleep in patients with KD. Even though sleep plays a crucial part in the neurodevelopment of children, the majority of research on sleep in children is concentrated on prevalent disorders, and it lags behind research conducted on sleep in adult populations [11]. Additionally, there is limited knowledge regarding the specific characteristics of sleep in patients with KD.

This study aimed to describe sleep disturbances and determine the relationship between these disturbances and clinical manifestations among KD patients.

## Patients and methods

### Study design and setting

This cross-sectional analytic study was carried out on 130 KD patients and 132 age- and sex-matched healthy controls. It was a survey-based study, and either the participants themselves or their parents were required to fill out an online questionnaire that was self-administered and prepared using Google Forms. The questionnaire was disseminated exclusively in the English language. To participate in the study, any KD patients from different countries who had been diagnosed with the disorder prior to reaching the age of 18 were eligible to participate. From the start of the study, patients who had been diagnosed with cancer or any chronic rheumatic, musculoskeletal, mental, or neurological disorder, have pre-existing sleep disturbances prior to the onset of KD were excluded from the study. KD patients at the acute attack were also excluded. Between the 6th of September 2019 and the 20th of March 2020, the questionnaire was sent out to all the potential participants in a random fashion using various social media platforms (including Facebook and WhatsApp). The potential participants were identified through web-based KD advertisements (Kawasaki disease Support, and Children with Aneurysms), provided that the patient was diagnosed by an expert pediatrician or rheumatologist. The verification mechanism involved a thorough review of the patient’s diagnostic reports. The participants were sent to a website that gave them instructions on how to fill out the questionnaire and supplied them with information regarding the purpose of the study being conducted. All the participants were given the assurance that their data would be kept confidential and that they would remain anonymous. The Google Form was directed to everyone who had consented to take part in the study. It was assumed that answering all the questions and thereafter submitting them constituted consent to participate in the study. Informed consent to participate was obtained from the parents or legal guardians of any participant under the age of 16. For the participants who were more than 16, we obtained their own consent.

The control group consisted of 132 healthy individuals. The control group consisted of age- and sex-matched healthy children with no history of KD or other inflammatory or cardiovascular conditions. These controls were selected to enable a clear comparison with KD patients. Matching was done to minimize confounding factors, such as differences in age or sex, that could influence the results. Additionally, children with recent febrile illnesses or chronic medical conditions were excluded to ensure the control group reflected a truly healthy population. The control group was recruited from pediatric outpatient clinics, community health centers, and well-child visits at local hospitals.

### Ethical consideration

This study was carried out in accordance with the principles outlined in the Helsinki Declaration [12]. The study protocol received approval from the Institutional Research Board of the Faculty of Medicine at Mansoura University, with the approval registration number R.24.07.2717.

### Sociodemographic characteristics and clinical data

Participants were asked questions about their age, gender, and whether the survey was completed by the patient or parent. Clinical data, including age of disease onset and disease duration, were obtained from KD participants.

The study also included the cumulative clinical data of patients with KD, which encompassed a medical history of prolonged high-grade fever lasting more than 5 days, as well as diagnoses of myocarditis, pericarditis, endocarditis, coronary artery disease, or chronic heart disease by experienced cardiologists. The participants were also queried about their history of conjunctivitis, uveitis, swollen and cracked lips, strawberry tongue, recurrent oral ulcers, swollen hands or feet, lethargy, irritability, joint inflammation, KD-related skin rash, desquamation of fingertips, abdominal pain due to KD, and chronic diarrhea. The participants were queried about whether they had ever received a diagnosis of macrophage activation syndrome from their physician.

### Therapeutic data

Therapeutic data was also collected from KD participants. The participants were interrogated regarding the medications they employed to manage their KD, such as IVIG, ASA, Infliximab, corticosteroids, or any other relevant medications.

### Children’s Sleep habits Questionnaire (CSHQ)

CSHQ is a validated questionnaire that evaluates the typical sleep patterns of preschool and school-age children. It consists of 33 items that measure sleep disturbances and three items that collect information about bedtime, wake-up time, and sleep length (nighttime sleep and daytime nap) during a “typical” recent week. In the case that the patients have an extraordinary event in the week before to the questionnaire, such as an examination, competition, or other similar occurrences, the parents are requested to fill out the questionnaire in accordance with the normative week that takes precedence. Using a Likert scale with three points, parents are asked to score the frequency of each item as follows: “usually” (five to seven times per week), “sometimes” (two to four times per week), and “rarely” (zero to one time per week). Higher scores suggest more frequent disturbances in sleep. The CSHQ provides a total sleep disturbance score and eight subscale scores, including bedtime resistance, sleep-onset delay, sleep duration, sleep anxiety, night wakings, parasomnias, sleep disorder breathing, and daytime sleepiness. A total score of ≥ 41 indicates clinically significant sleep disturbance [13].

### Statistical analysis

The data analysis was conducted using the Statistical Package for Social Science (SPSS) program version 22. The presentation of quantitative data consisted of means with standard deviations (SD) for parametric variables and medians (min.-max.) for nonparametric variables. On the other hand, qualitative data was expressed as percentages and numbers. The Shapiro-Wilk test was implemented to ascertain the normality of the variable distribution. The independent samples t-test was employed to evaluate the significance of differences between two groups for normally distributed data, while the Mann-Whitney test was employed for non-parametric variables. Chi-square or Fisher exact tests were implemented as appropriate for qualitative variable comparisons. Logistic regression analysis was implemented to evaluate the correlation between clinical and therapeutic data and the CSHQ subscale and total scores of KD patients, employing the enter approach. Statistical significance was defined as a p value of less than 0.05.

## Results

**Fig. 1:**
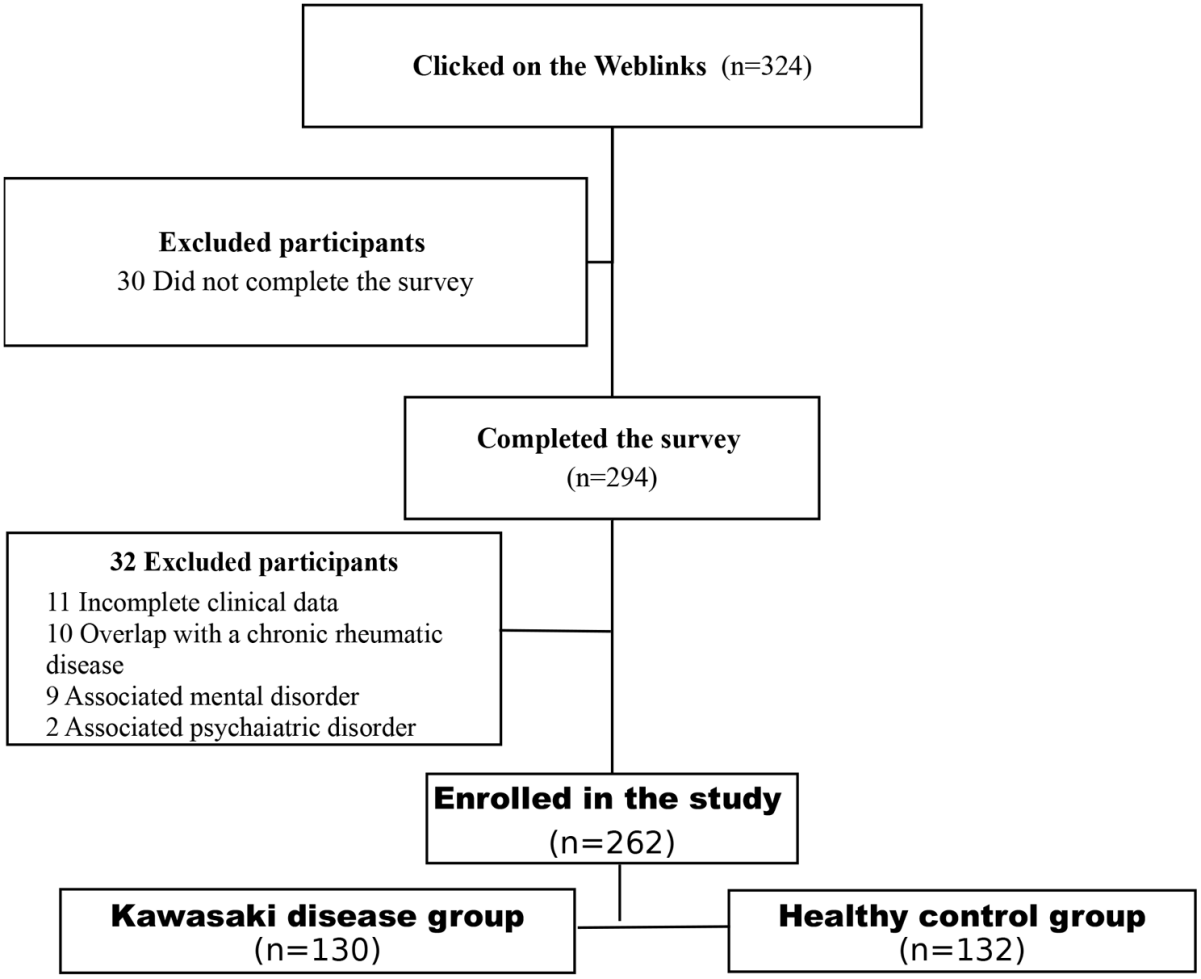
The flowchart of the study

During the study, there were 324 participants who clicked on the weblinks that sent them to the online survey, but only 294 of them completed it. Thirty-two of them were excluded for a variety of reasons, including the fact that eleven had incomplete clinical data, ten had overlapping chronic rheumatic disease, nine had an associated mental disorder, and one had a psychiatric disorder. Participants were assigned to two groups: KD and healthy control, as shown in Fig. 1.

The study included 130 KD participants and 132 healthy controls. The median age for KD patients was 6 years, and 51.5% of them were female. The healthy controls were matched for age and sex (median age 6.5 years, 53% female).

In the KD group, the median age at disease onset was 2.5 years, and the median disease duration was 3 years. The most reported manifestations of KD were persistent high-grade fever (93.1%), KD-related skin rash (86.9%), swollen and cracked lips (83.1%), lethargy and irritability (83.1%), strawberry tongue (73.8%), and non-purulent conjunctivitis (70.0%). Swollen feet (65.4%) and hands (62.3%) were also frequently reported. Coronary artery disease was observed in approximately 25.4% of the patients, while macrophage activation syndrome was only present in 2 patients, accounting for 1.5%. Regarding treatment, IVIG was the most administered medication (90.8%), followed by ASA (86.9%). Other clinical and therapeutic data were illustrated in Table 1.

**Table 1:** Study population demographic and clinical characteristics at baseline

Variable *n* (%), median (min-max)	Kawasaki patients (*n* = 130)
**Demographic data**	
Age, years	6.0 (0.58-39)
Gender	
Female Male	63 (48.5) 67 (51.5)
The survey completed by	
The patient The parent	5 (3.8) 125 (96.2)
Country	
East Asian countries North America Europe Australia and South America Egypt Others	28 (21.5) 26 (20.0) 24 (18.5) 20 (15.4) 10 (7.7) 22 (16.9)
**Clinical data**	
Age at disease onset, years	2.5 (0.04-14)
Disease duration, years	3 (1–36)
**Cumulative manifestations**	
High fever > 5 days	121 (93.1)
Myocarditis	6 (4.6)
Pericarditis	3 (2.3)
Endocarditis	4 (3.1)
Coronary artery disease	33 (25.4)
Chronic heart disease	1 (0.8)
Conjunctivitis	91 (70.0)
Uveitis	15 (11.5)
Swollen and cracked lips	108 (83.1)
Strawberry tongue	96 (73.8)
Recurrent oral ulcers	22 (16.9)
Swollen hands	81 (62.3)
Swollen feet	85 (65.4)
Lethargy, irritability	108 (83.1)
Arthritis	32 (24.6)
Skin rash	113 (86.9)
Desquamation of fingertips	44 (33.8)
Abdominal pain	61 (46.9)
Chronic diarrhea	27 (20.8)
Macrophage activation syndrome	2 (1.5)
**Therapeutic data**	
Intravenous immunoglobulin (IVIG)	118 (90.8)
Acetylsalicylic acid	113 (86.9)
Infliximab	4 (3.1)
Corticosteroids	24 (18.5)
Anakinra	4 (3.1)
Cyclosporine	1 (0.8)
Clopidogrel	2 (1.6)
Warfarin	1 (0.8)

*p < 0.05

The sleep scores of patients with KD were significantly higher than those of the control group (55.72 ± 11.97 vs. 49.45 ± 8.54, *p* < 0.001). The total duration of sleep did not exhibit any statistically significant difference between patients with KD and healthy controls (*p* = 0.399). Table 2 illustrates the comparison of subscale scores between KD patients and healthy controls. KD patients exhibited significantly elevated scores in sleep onset delay (*p* < 0.001), sleep duration (*p* < 0.001), night wakings (*p* < 0.001), parasomnias (*p* < 0.001), and sleep-disordered breathing (*p* < 0.001). Additionally, they showed marginally higher scores in daytime sleepiness (*p* = 0.059) compared to healthy controls.

**Table 2:** CSHQ subscale and total scores for Kawasaki patients (*n* = 130) and healthy controls (*n* = 132)

Variable *n* (%), median (min-max), mean ± SD	KD group (*n* = 130)	Healthy control group (*n* = 132)	*P*
Average bedtime, mean	20:18	19:40	< 0.001*
Average waketime, AM, mean	7:22	9:05	< 0.001*
Total sleep duration, hr	10 (2-15.5)	10 (5–15)	0.399
Bedtime Resistance	10.74 ± 3.73	11.33 ± 3.21	0.168
Sleep Onset Delay	1.93 ± 0.84	1.59 ± 0.065	< 0.001*
Sleep Duration	4.94 ± 1.88	3.74 ± 1.14	< 0.001*
Sleep Anxiety	7.69 ± 2.70	7.74 ± 2.55	0.878
Night Wakings	5.48 ± 2.04	4.59 ± 1.57	< 0.001*
Parasomnias	11.84 ± 2.70	8.91 ± 1.94	< 0.001*
Sleep-Disordered Breathing	4.13 ± 1.59	3.45 ± 0.88	< 0.001*
Daytime Sleepiness	12.98 ± 3.62	12.23 ± 2.72	0.059
Total score	55.72 ± 11.97	49.45 ± 8.54	< 0.001*

*p < 0.05

Table 3 illustrates the correlation between clinical and therapeutic data and sleep scores in KD patients. Younger age was associated with higher rates of bedtime resistance (*p* < 0.001) and sleep anxiety (*p* = 0.005). Younger age at disease onset was associated significantly with higher rates of bedtime resistance (*p* = 0.009), sleep anxiety (*p* = 0.038), night wakings (*p* = 0.017), and worse sleep quality (*p* = 0.033). Endocarditis was associated with higher rates of parasomnias (*p* = 0.028), sleep-disordered breathing (*p* = 0.016), and worse sleep quality (*p* = 0.045). Lethargy and irritability were associated with longer sleep duration (*p* = 0.003), higher rates of night wakings (*p* = 0.024), parasomnias (*p* = 0.033), and worse sleep quality (*p* = 0.030). Regarding therapy, corticosteroids were associated with higher rates of night wakings (*p* = 0.007), parasomnias (*p* = 0.036), and worse sleep quality (*p* = 0.013).

**Table 3:** Correlation between clinical and therapeutic data and sleep quality scores in Kawasaki patients (*n* = 130)

Variable	Bedtime Resistance		Sleep Onset Delay		Sleep Duration		Sleep Anxiety		Night Wakings		Parasomnias		Sleep-Disordered Breathing		Daytime Sleepiness		Total CSHQ score	
	OR	*p*	OR	*p*	OR	*p*	OR	*p*	OR	*p*	OR	*p*	OR	*p*	OR	*p*	OR	*p*
Age, years	-0.310	< 0.001*	0.049	0.587	0.113	0.210	-0.251	0.005*	-0.164	0.067	-0.011	0.901	-0.106	0.239	0.320	< 0.001*	-0.037	0.679
Age at disease onset	-0.229	0.009*	0.052	0.559	-0.106	0.230	-0.182	0.038*	-0.208	0.017*	-0.093	0.291	-0.073	0.410	-0.085	0.337	-0.188	0.033*
Disease duration	-2.657	0.009*	0.041	0.646	0.171	0.057*	-0.184	0.040*	-0.070	0.440	0.049	0.587	-0.055	0.539	0.392	< 0.001*	0.059	0.513
**Cumulative manifestations**																		
High fever > 5 days	-0.068	0.441	0.086	0.328	0.007	0.935	0.081	0.358	0.065	0.463	-0.005	0.954	0.099	0.261	-0.052	0.555	0.019	0.831
Myocarditis	-1.057	0.293	-0.114	0.198	0.007	0.935	-0.084	0.344	-0.016	0.854	0.000	0.996	-0.088	0.321	0.164	0.062	-0.007	0.937
Pericarditis	0.052	0.556	0.074	0.401	-0.077	0.384	0.037	0.680	0.114	0.195	0.124	0.161	0.149	0.090	0.072	0.415	0.107	0.227
Endocarditis	0.108	0.220	0.068	0.441	0.053	0.546	0.120	0.176	0.089	0.315	0.193	0.028*	0.211	0.016*	0.075	0.394	0.176	0.045*
Coronary artery disease	0.041	0.643	0.006	0.946	0.000	0.997	-0.038	0.665	0.087	0.325	0.068	0.442	0.086	0.331	0.092	0.298	0.081	0.362
Chronic heart disease	0.125	0.158	0.113	0.201	0.144	0.102	0.108	0.221	0.152	0.084	0.136	0.122	-0.007	0.934	0.147	0.095	0.179	0.041*
Conjunctivitis	-0.177	0.044*	0.006	0.946	0.014	0.871	-0.075	0.398	-0.092	0.300	-0.046	0.606	0.065	0.464	-0.027	0.756	-0.072	0.416
Uveitis	0.019	0.831	0.145	0.099	0.102	0.249	-0.030	0.733	0.091	0.301	-0.041	0.643	0.001	0.995	0.009	0.919	0.037	0.677
Swollen and cracked lips	-0.004	0.963	0.036	0.681	-0.048	0.590	0.063	0.479	-0.003	0.969	-0.035	0.694	0.011	0.898	0.014	0.873	0.006	0.942
Strawberry tongue	-0.047	0.599	0.161	0.068	0.130	0.140	0.049	0.580	0.021	0.810	0.082	0.357	-0.006	0.945	0.176	0.045*	0.108	0.223
Recurrent oral ulcers	-0.007	0.938	-0.012	0.895	0.103	0.246	-0.040	0.653	0.044	0.621	0.081	0.362	0.105	0.233	0.088	0.318	0.080	0.368
Swollen hands	-0.136	0.123	0.069	0.437	0.025	0.775	-0.030	0.736	-0.018	0.843	0.048	0.589	-0.016	0.857	0.057	0.522	0.000	0.999
Swollen feet	-0.138	0.118	0.057	0.519	-0.027	0.765	-0.026	0.768	-0.039	0.662	0.052	0.560	0.001	0.987	0.033	0.709	-0.020	0.822
Lethargy, irritability	0.012	0.889	0.135	0.126	0.259	0.003*	0.032	0.716	0.198	0.024*	0.187	0.033*	0.115	0.192	0.105	0.233	0.190	0.030*
Arthritis	-0.186	0.035*	-0.060	0.500	0.086	0.333	-0.134	0.130	-0.048	0.585	0.081	0.360	0.616	0.539	0.167	0.057*	0.018	0.838
Skin rash	0.034	0.700	0.077	0.382	-0.110	0.212	0.049	0.581	-0.042	0.634	0.019	0.829	0.119	0.179	-0.072	0.415	-0.007	0.934
Desquamation of fingertips	-0.042	0.639	0.157	0.075	0.136	0.122	0.015	0.863	0.085	0.335	0.146	0.098	0.033	0.706	0.050	0.574	0.097	0.270
Abdominal pain	-0.083	0.347	0.189	0.031*	0.138	0.118	0.050	0.571	0.087	0.327	0.183	0.037*	0.186	0.035*	0.109	0.218	0.141	0.109
Chronic diarrhea	0.118	0.183	-0.026	0.771	0.078	0.380	0.087	0.327	0.027	0.759	0.087	0.323	0.090	0.311	0.161	0.067	0.141	0.110
Macrophage activation syndrome	0.143	0.104	0.160	0.068	0.138	0.118	0.177	0.044	0.185	0.035*	0.240	0.006*	0.069	0.437	0.122	0.166	0.228	0.009*
**Therapeutic data**																		
Intravenous immunoglobulin (IVIG)	0.099	0.262	0.026	0.765	-0.167	0.058*	0.072	0.415	-0.029	0.747	-0.059	0.507	-0.007	0.935	-0.135	0.126	-0.059	0.506
Acetylsalicylic acid	0.041	0.648	-0.007	0.939	-0.025	0.779	-0.022	0.808	0.007	0.941	0.067	0.452	0.093	0.297	0.161	0.068	0.078	0.377
Infliximab	-0.035	0.689	-0.092	0.298	-0.042	0.637	-0.112	0.205	-0.064	0.467	-0.056	0.530	0.042	0.638	-0.073	0.409	-0.074	0.402
Corticosteroids	0.135	0.126	0.063	0.474	0.132	0.134	0.157	0.074	0.237	0.007*	0.184	0.036*	0.086	0.330	0.124	0.159	0.217	0.013*

*p < 0.05

There were 108 patients (83.1%) who reported lethargy and irritability. The patients with and without lethargy and irritability were not significantly different regarding scores of bedtime resistance (*p* = 0.881), sleep onset delay (*p* = 0.136), and sleep anxiety (*p* = 0.757). Patients with lethargy and irritability had statistically significant higher scores of sleep duration (*p* = 0.002), night wakings (*p* = 0.027), parasomnias (*p* = 0.039), and total sleep scores (*p* = 0.039) as shown in Table 4.

**Table 4:** Subscale comparisons between Kawasaki disease patients without and with lethargy and irritability

Variable median (min-max)	Lethargy and irritability		*p*
	Without (*n* = 22)	With (*n* = 108)	
Average bedtime, mean	20:00	20:21	1
Average waketime, AM, mean	7:32	7:20	0.188
Total sleep duration, hr	10.75 (6–15)	10 (2-15.5)	0.129
Bedtime Resistance	10.50 (6–17)	10 (6–18)	0.881
Sleep Onset Delay	2 (1–3)	2 (1–3)	0.136
Sleep Duration	3 (3–7)	5 (3–9)	0.002*
Sleep Anxiety	7 (4–12)	8 (4–12)	0.757
Night Wakings	4.5 (3–8)	6 (3–9)	0.027*
Parasomnias	11 (8–14)	12 (7–20)	0.039*
Sleep-Disordered Breathing	3 (3–8)	3 (3–9)	0.312
Daytime Sleepiness	11 (8–20)	13 (8–23)	0.228
Total score	47.5 (36–65)	58 (33–80)	0.039*

*p < 0.05

## Discussion

The results of this study indicated that patients with KD had more sleep problems than their healthy peers. When compared to healthy controls, patients with KD reported higher sleep onset delay, sleep duration, night wakings, parasomnias, sleep-disordered breathing, daytime sleepiness, and total sleep scores. To the best of our knowledge, this is the first study investigating sleep patterns and disturbances and possible associated factors associated with sleep disturbances in KD patients. As a result, there is no other study available to compare with our findings.

Sleep provides the body with the opportunity to save energy, replenish its natural functions, encourage physical growth, and support mental development. Sleep also helps the body to grow physically [14]. Indeed, inadequate sleep quality and duration have a significant impact on physical, cognitive, and behavioral development, leading to delayed growth and poor emotional control [15]. Patients with rheumatic and immunological diseases are at an increased risk of developing sleep disorders, and over 75% of these patients report experiencing sleep disturbances [16]. This may be related to increased proinflammatory signals in both the peripheral and central nervous systems [17]. Clinical research has focused on many aspects of sleep habits, such as duration, timing, quality, and presence of disturbances [18]. We used the CSHQ for assessment of sleep quality in KD patients as it is one of the most frequently utilized parent-reported questionnaires for investigating sleep habits in children [13]. In our cohort, the sleep quality of patients with KD was significantly worse than that of the control group.

It was acceptable to find that KD patients with younger ages were associated with poorer sleep quality. In a study involving 4314 participants, the authors found that younger age groups experienced an increase in certain sleep disturbances, such as difficulties falling asleep, anxiety before bedtime, nightmares, and sleep terrors [19].

KD is the most common cause of acquired heart disease in children in developed countries [20]. It is characterized by abnormalities in the coronary artery, which can lead to myocardial ischemia, infarction, and potentially fatal outcomes [21]. While valvular endocardium can be affected by KD, mitral valvulitis is often mild and transient [22]. Only 4 patients in our study reported endocarditis, which was associated with worse sleep quality. Approximately 25.4% of our KD patients reported having coronary artery disease. Coronary artery abnormalities are common complications of KD, with 15–25% of children developing aneurysms if not treated [23]. The frequency of coronary artery lesions decreased from 25 to 5% following therapy with IVIG plus ASA [24]. Most recorded instances of infarction occur during periods of sleep or while in a state of rest [25]. In our KD cohort, coronary artery disease did not appear to be associated with sleep scale or subscale scores. In this regard, a study of 89 patients with KD found no correlation between coronary artery disease and health related quality of life [26].

In the current study, KD Patients who developed chronic heart disease had significantly worse sleep scores than those who did not. Patients with chronic heart disease often experience sleep problems such as short sleep duration, poor sleep quality, and breathing disorders [27]. In addition, insufficient or poor sleep is a significant risk factor for cardiovascular disease [28]. A review of experimental and clinical data suggests that inadequate sleep can lead to cardiovascular disease through changes in autonomic, metabolic, endothelial, coagulation, and inflammation functions [29]. These changes can affect eating behaviors and hormone levels in children and adolescents [30]. Additionally, coronary artery abnormalities cause sleep disturbance by creating discomfort and anxiety, which can lead to restless nights and poor sleep patterns. Moreover, the physical toll of heart-related issues often exacerbates fatigue, creating a vicious cycle that further complicates both sleep and cardiovascular health [31].

KD can also cause different ocular symptoms, including bilateral conjunctivitis [32]. The quality of sleep of children may be impacted by chronic eye irritation caused by conjunctivitis [33]. About two-thirds of our cohort reported non-purulent conjunctivitis. However, there was no significant association between conjunctivitis and sleep disturbances. Uveitis is the prevailing ocular manifestation in KD [34]. According to Burns et al., 83% of children diagnosed with KD develop uveitis within a week [35]. Uveitis is often mild and bilateral, and it may be a valuable indicator in diagnosing incomplete KD [34]. A uveitis attack may be precipitated by stress and inadequate sleep [36]. In the present study, 11.5% of KD patients reported uveitis, with no significant association with sleep disturbances.

Neurologic involvement in KD is reported in 1.1–3.7% of cases. It ranges from mild neurologic symptoms, such as irritability and lethargy, to CNS involvement [37]. An unexplained high fever and marked irritability or lethargy may be the only initial findings in very young infants [38]. Lethargy and irritability were prevalent in our KD cohort, with a significant association with worse sleep quality.

In the present study, about one fifth of KD patients reported corticosteroids administration, which was associated with higher rates of night wakings, parasomnias, and poor sleep quality. Corticosteroids are beneficial in treating vasculitis due to their potent anti-inflammatory properties. Soon after KD was identified, its potential use was identified, and it was anticipated to be beneficial [39]. In the acute stage of KD, the combination of IVIG and corticosteroids can rapidly decrease cytokine levels within a 24-hour period [40]. Corticosteroid use may increase the risk of developing coronary artery lesions; however, a causal association cannot be established [41].In real-world drug usage cases, 7.7 to 18% of an American cohort took corticosteroids following unsuccessful IVIG treatment [42]. Additionally, 14.5% of a Spanish cohort got corticosteroids as primary or secondary treatment [43]. Sleep disturbances are a commonly overlooked adverse effect of steroids. Insomnia, a type of sleep disturbance, is included as an adverse neurological response in the FDA’s labeling for corticosteroids, along with euphoria, mood swings, and severe depression [44]. According to a study evaluating self-reported frequency of steroid-induced adverse reactions, sleep disturbance was among the most often mentioned side effects, claimed by up to 60% of patients [45].

This study has certain limitations that require attention. One limitation of this study is that we analyzed the patient- and parent-reported measures together, regardless of who completed the questionnaires. We conducted this analysis regardless of who completed the questionnaires. Even though this combination maximizes the total sample size, there may be differences in perceptions between children and proxies, particularly for children who are older. It is necessary to evaluate our sleep scores with respect to the treatments that were administered to the children who were included in this cohort. One more thing to consider is that the recruitment of participants through social media could potentially result in biassed outcomes. The number of patients with KD is, to a certain extent, restricted, and the utilization of this approach could potentially make it easier to enroll enough KD patients. KD patients were evaluated during the follow up period which might lead to recall bias. Finally, our findings support the need for further research to better understand the nature and etiology of sleep problems and identify the most effective treatment modalities in KD patients.

In conclusion, patients with KD experience higher sleep disturbance than their healthy counterparts. Lethargy and corticosteroid use are linked to poor sleep quality. This study emphasizes the need of evaluating sleep disturbances in KD patients.

## Data Availability

The datasets used and/or analysed during the current study are available from the corresponding author on reasonable request.

## Abbreviations

ASA: Aspirin
CSHQ: Children’s Sleep Habits Questionnaire
IVIG: Intravenous immune globulin
KD: Kawasaki disease
